# Objectively-Assessed Patterns and Reported Domains of Sedentary Behavior Among Japanese Older Adults

**DOI:** 10.2188/jea.JE20180041

**Published:** 2019-09-05

**Authors:** Ai Shibata, Koichiro Oka, Kaori Ishii, Rina Miyawaki, Shigeru Inoue, Takemi Sugiyama, Neville Owen

**Affiliations:** 1 Faculty of Health and Sport Sciences, University of Tsukuba, Ibaraki, Japan; 2 Faculty of Sport Sciences, Waseda University, Saitama, Japan; 3 School of Arts and Letters, Meiji University, Tokyo, Japan; 4 Department of Preventive Medicine and Public Health, Tokyo Medical University, Tokyo, Japan; 5 Mary MacKillop Institute for Health Research, Australian Catholic University, Melbourne, VIC, Australia; 6 Behavioural Epidemiology Laboratory, Baker Heart and Diabetes Institute, Melbourne, VIC, Australia; 7 Swinburne University of Technology, Hawthorn, VIC, Australia

**Keywords:** prolonged sitting, breaks, accelerometer, elderly

## Abstract

**Background:**

Prolonged periods of sitting time can be adversely associated with older adults’ well-being and functional capacities. Understanding patterns and contexts of sedentary behaviors (SB) can inform approaches to prevention. This study examined Japanese older adults’ objectively-assessed patterns and reported domains of SB and their interrelationships.

**Methods:**

Participants (*n* = 297; aged 65–84 years) of this cross-sectional study wore an accelerometer for 7 days and completed a survey. Five measures related to SB patterns were identified from the accelerometer data. SB from six domains, socio-demographics, and chronic conditions were identified from the survey data. Relative contributions of six domains to objectively-measured prolonged sedentary time (≥30 minutes) and the number of breaks were examined in a series of multivariate linear regressions. Covariates were socio-demographics, chronic conditions, and accelerometer wear time.

**Results:**

On average, participants spent 8.8 hours a day sedentary (58% of accelerometer wear time), with 7.6 breaks per sedentary hour, and 3.7 hours a day through prolonged sedentary bouts (4.4 time/day). The proportions of time in the SB domains were 9.4% for car, 4.0% for public transport, 6.1% for work, 45.5% for television (TV) viewing, 9.8% for computer use, and 25.1% for other leisure. Domains of SB that contributed significantly to longer sedentary time through prolonged bouts were TV viewing and computer use. TV viewing was also associated with a lesser number of breaks.

**Conclusions:**

For Japanese older adults, initiatives to address SB could focus on breaking-up prolonged periods of SB by encouraging more frequent breaks, especially during TV viewing.

## INTRODUCTION

Sedentary behavior, which is distinct from not engaging in physical activity, is a known health risk^1^ and is prevalent among older adults.^2^ Greater time spent in total sedentary behavior and/or television (TV) viewing time has been shown to be associated adverse health outcomes^3^ and with poorer cognitive and functional capacities among older adults.^4^^,^^5^ In addition to the duration of sedentary behavior, its pattern (how it is accumulated through shorter or longer bouts) is also related to health outcomes such as reduced risk of impairment in activities of daily living and physical function.^6^^,^^7^ Furthermore, sedentary behavior in different domains (eg, transportation, TV viewing, or computer use) may not be equally related to health outcomes; for instance, mentally-active sedentary behavior, such as computer use and reading books, was found to be beneficially associated with cognitive function in older adults.^4^^,^^8^ It is possible that sedentary behaviors in different domains vary in their patterns (eg, length of bouts), which may produce differential impacts on health outcomes.

A small number of recent studies in the United States, the United Kingdom, and Belgium have examined patterns of sedentary behavior among older adults.^9^^–^^11^ However, patterns of sedentary behavior among older adults in non-Western countries have not been reported. Given that there are between-country differences in time spent sedentary^12^ and the different genetic and environmental profiles (eg, lifestyles, culture, or social system) associated with health problems in Western countries,^13^ it is important to understand how sedentary behavior is accumulated in non-Western countries. Evidence on Japanese older adults is of particular importance, because Japan has a high average life expectancy and rapid aging of the population compared to other countries.

For domains of sedentary behavior, most previous studies on older adults’ sedentary behavior have focused on total sedentary time or on sedentary time in one specific domain (eg, TV viewing or computer use).^14^^–^^18^ A recent Japanese study and a Taiwanese study reported time spent in specific leisure-time sedentary behaviors, such as TV viewing, computer and internet use, or reading books or newspapers.^19^^,^^20^ However, little is known about non-leisure time sedentary behaviors, including those for transport or working. In addition, no research appears to have examined how different domains of sedentary behavior contribute to the patterns of sedentary behavior, such as time spent in prolonged bouts of sitting and the number of breaks. A Belgian study examined both objectively-measured total sedentary time and three domains of sedentary behavior (driving a car, computer use, and TV viewing) but did not conduct any analysis linking these two measures.^21^ Thus, how older adults accumulate or break sedentary time, and in which contexts, is not well known.

The present study examined the patterns and domains of sedentary behavior among older Japanese adults and what domains of sedentary behavior contributed to prolonged bouts and breaks in sedentary time.

## METHODS

### Participants and procedure

Participants of this cross-sectional study were from Matsudo, a suburban city east of Tokyo (population: approx. 0.5 million). Of the 107,928 community-dwelling older adults aged 65–84 years living in this city as of April 2013, 3,000 were randomly selected from the paper-based registry of residential addresses, stratified by gender and age (65–74 years and 75–84 years). The study recruited only one adult from each household. If two or more adults were selected from one household, we retained the person who was chosen first and replaced the other using further sampling from the equivalent category of gender and age. The study was conducted in 2013 and involved two phases of data collection: a self-administered postal survey and on-site examinations. The postal surveys were mailed to 3,000 potential participants two weeks after posting invitation letters. The response rate for the survey was 42% (*n* = 1,250). Items such as sociodemographic attributes, exercise habit, and chronic conditions were obtained in the survey. A 500-yen book voucher was offered to each participant who completed the postal survey. Responders of the postal survey were also asked to indicate whether or not they could receive an invitation letter for a future additional survey of this study. Those who accepted our request (*n* = 951) were provided with formal invitation letters for on-site examination via postal mail. At the testing site, height and weight of participants were measured and they were then asked to complete a domain-specific sedentary behavior questionnaire, wear the accelerometer, and record an activity log for 7 consecutive days. A 1000-yen book voucher was offered to each participant who completed this process.

We aimed to collect accelerometer data from 250 or more participants, based on the sample size of recent previous studies examining older adults’ physical activity and sedentary behavior using the accelerometer device. We projected that the response rate to postal surveys for this age group would be around 30–40% and that 70% of respondents would not meet the inclusion criteria (ie, attending on-site examination, wearing accelerometer for 7 days). We, thus, estimated that we needed to contact at least 2,500 people (≈ 250/0.35/0.3) to achieve the target.

The final sample of this study consisted of those who completed the postal survey and the on-site examinations (*n* = 330). Those included in the final study sample were significant more likely to be married and to be physically active than were those who accepted the invitation to take part in the further examination after the postal survey but who did not complete that on-site examination (*n* = 621); there were no significant differences in age, educational attainment, and number of chronic conditions. Written informed consent was obtained from all participants. Of these, those who had insufficient accelerometer data (*n* = 30) or missing data for relevant variables (*n* = 16) were excluded (numbers not mutually exclusive). The final sample size was 287. The study was approved by the Waseda University Ethics Committee (2013-265).

### Measures

#### Objectively measured sedentary behavior

Participants were asked to wear a tri-axial accelerometer (Active style Pro HJA 350-IT; Omron Healthcare Co. Ltd., Kyoto, Japan) on the left hip during waking hours for 7 consecutive days. Intensity of activity by metabolic equivalents (METs), determined using built-in algorithms of this accelerometer, have been reported to be closely correlated with METs calculated using the indirect calorimetry.^22^ Data were recorded in 1-minute epochs. A comparative study of activity monitors showed that this accelerometer underestimated total time spent in sedentary behavior (−25.6 min/day) compared to the activePAL3 as the criterion.^23^ Non-wear time was defined as intervals of at least 60 consecutive minutes of no activity (estimated intensity of 0.9 or less METs) based on a validity study,^22^ with allowance for up to 2 min of some limited movement (≤1.0 METs). Days with at least 10 h of wear time were considered valid. Participants with at least 4 valid days, including at least 1 weekend day, were included in the analyses. Five sedentary behavior measures were derived: mean daily total sedentary time (min/day), proportion of sedentary time (% of wear time), number of breaks (times/sedentary hour), number of prolonged bouts (times/day), and proportion of sedentary time through prolonged bouts (% of total sedentary time). Sedentary time was defined as a period of any activity with an intensity of ≤1.5 METs. A sedentary bout was defined as a period of uninterrupted sedentary time.^24^ A prolonged sedentary bout was defined as at least 30 consecutive minutes of sedentary time.^25^ A break in sedentary behavior was defined as at least 1 minute of non-sedentary bout in between two sedentary bouts.^24^ Mean weekday and weekend values for total sedentary time, number of breaks, and number and total of prolonged bouts were first calculated on each valid weekday and weekend day. Then, mean daily total values of these measures were computed by weighting for 5 weekdays and 2 weekend days. Next, further summary measures were calculated: proportion of sedentary time in wear time; number of breaks per sedentary hour (mean daily number breaks/mean daily total sedentary time in hour); and proportion of prolonged sedentary time to total sedentary time.

#### Self-reported sedentary behavior in different domains

Participants were asked to report daily average time spent in sedentary behavior in hours and minutes over the past 7 days for the following six domains: while riding in a car as driver or passenger; using public transport; at work; watching television, videos, and digital video discs (DVDs); using a computer, cell phone, and tablet personal computer (PC) for non-work purposes; and sitting for other purposes in leisure time (eg, talking, reading, listening to music, or engaging in hobby). They were asked to provide separate response for workdays (or weekdays for unemployed individuals) and non-workdays (weekends). Mean workday and non-work day values of total sedentary time were calculated by summing all six domains separately for workdays and non-workdays. Mean daily values of total sedentary time and each domain’s sedentary time were also calculated by weighting for number of workdays and non-work days. This instrument was reported to have fair to good validity for estimating total sedentary time against objectively-measured sedentary time using accelerometer and to be reliable among Japanese adults aged 40–64 years.^26^

#### Sociodemographic attributes and chronic conditions

The postal survey asked participants to report gender, age, educational attainment (university or further education; high school or less), marital status (currently married; single), and the presence of chronic conditions (stroke, cardiovascular diseases, diabetes, dyslipidemia, hyperuricemia, peripheral vascular disease, osteoporosis, knee osteoarthritis, hip osteoarthritis, spondylosis, spinal canal stenosis, rheumatoid arthritis, collagenosis, cancer). The reported number of chronic conditions was categorized into none, 1, and 2 or more. Body mass index (BMI; kg/m^2^) was calculated from the height and weight measured at the testing site and classified as normal weight (<25 kg/m^2^) or overweight (≥25 kg/m^2^).

### Statistical analyses

Overall mean values of five objective patterns and six self-reported domain-specific measures of sedentary behavior were identified. Differences in the measures of objective and self-reported time spent in sedentary behavior between subgroups were examined by adjusting for other socio-demographic variables (and wear time only for objective measures). In addition, the relative contributions of the six sedentary domains (10-min increment) to objectively-measured prolonged sedentary time (minutes) and the number of breaks (per sedentary hour) were examined via a series of multivariate liner regressions using prolonged sedentary time or breaks as the outcome. Covariates were gender, age, BMI, marital status, educational status, and accelerometer-wear time, and the number of chronic conditions. Both unadjusted and adjusted models were analyzed. Analyses were conducted using STATA 15.0 (StataCorp LLC, TX, USA). Statistical significance was set at a two sided *P*-value of <0.05.

## RESULTS

Table 1 shows the characteristics of study participants and time spent in sedentary behavior. Of the study sample, 26.3% (*n* = 73) reported working full-time or part-time. The mean age of participants was 74.5 (standard deviation [SD], 5.2) years. They wore the accelerometer for a mean of 15.0 (SD, 1.4) hours per day over a mean of 7.2 (SD, 0.9) valid wearing days. Overall, participants spent 8.8 hours (or 58% of wear time) per day sedentary. Daily total sedentary time was significantly higher in men, the older age group, and those who were overweight. The mean number of breaks was 7.6 (SD, 2.9) per sedentary hour. The number of breaks was higher in women and those who were normal weight. About two-fifths of the time spent in sedentary behavior was accumulated by prolonged bouts that lasted 30 minutes or longer. The number of prolonged sedentary bouts and proportion of sedentary time accumulated in prolonged bouts were significantly higher in men and those who were overweight.

**Table 1.** Patterns of objectively-measured sedentary behavior

	*n*	(%)	Daily total SB^a^		Proportion of SB^b^		Number of breaks^c^		Number of prolonged bout^d^		Proportion of SB through prolonged bout^e^	
				
			Mean	SD	Mean	SD	Mean	SD	Mean	SD	Mean	SD
**All participants**	287	100	525.2	112.3	58.3	11.7	7.6	2.9	4.4	1.9	42.2	14.6
**Gender**												
Men	177	61.7	550.2	116.3^***^	61.8	11.3^***^	7.1	2.9^***^	4.9	1.9^***^	44.8	14.5^**^
Women	110	38.3	484.8	92.7	52.7	10.1	8.6	2.6	3.8	1.7	38.1	14.0
**Age group, years**												
65–74	143	49.8	511.3	112.9^*^	55.8	11.4^*^	8.0	3.1	4.2	1.9^*^	40.1	14.6
75–84	144	50.2	538.9	110.4	60.8	11.5	7.3	2.6	4.7	1.9	44.4	14.3
**Educational attainment**												
≤high school	175	61.0	516.5	116.4	57.4	12.2	7.7	2.6	4.3	1.9	41.5	15.0
≥university	112	39.0	538.6	104.7	59.9	10.9	7.6	3.3	4.7	1.8	43.4	13.9
**Marital status**												
single	51	17.8	541.6	121.7	60.0	11.9	7.6	2.7	4.5	2.0	42.6	14.4
married	236	82.2	521.6	110.1	58.0	11.7	7.6	2.9	4.4	1.9	42.2	14.7
**BMI, kg/m^2^**												
<25	198	69.0	513.7	110.1^**^	56.6	10.7^**^	8.0	3.0^*^	4.2	1.9^**^	39.8	13.9^***^
≥25	89	31.0	550.6	113.7	62.2	13.1	6.8	2.4	5.0	1.9	47.6	14.7
**Chronic conditions**												
0	75	26.1	515.5	118.4	57.1	11.6	8.1	2.7	4.2	1.9	39.7	15.1
1	102	35.5	517.3	104.9	57.7	11.1	7.7	3.3	4.4	1.8	43.0	13.6
≥2	110	38.3	539.1	114.3	59.8	12.4	7.3	2.6	4.6	1.9	43.3	15.1

BMI, body mass index; SB, sedentary behavior; SD, standard deviation.

^a^min/day.

^b^% of wear time.

^c^times/sedentary hour.

^d^times/day.

^e^% of total SB.

Using multivariate linear regression, the differences in each objective measures of sedentary behavior between subgroups of each sociodemographic variable were examined with two-sided test.

^***^P < 0.001; ^**^P < 0.01, ^*^P < 0.05.

Self-reported time spent in sedentary behavior for each domain and sub-group differences are shown in Table 2. On average, the participants reported 7.2 (SD, 3.5) hours a day in sedentary behavior. The Pearson correlation coefficient between objective and self-reported total time spent in sedentary behavior was 0.28 (*P* < 0.001). There were no significant sociodemographic differences in total sedentary time, but it was higher among those who were overweight. The proportion of time spent sedentary for each of the six domains was 9.4% for car, 4.0% for public transport, 6.1% for work, 45.5% for TV viewing, 9.8% for computer use, and 25.1% for other leisure. Sedentary behavior during public transport use was significantly higher in women and those who were overweight. Those who were single had a significantly longer time on TV viewing than those who were married. Sedentary time for computer use was longer in men and those with university degree or higher. Those who were overweight had a longer sedentary time on other leisure purposes.

**Table 2.** Duration of domain-specific sedentary behaviors (minutes/day)

	Total		Car		Public Transport		Work		TV		PC		Other leisure	
						
	Mean	SD	Mean	SD	Mean	SD	Mean	SD	Mean	SD	Mean	SD	Mean	SD
**All participants**	431.2	209.1	40.7	72.1	17.2	29.8	26.3	76.5	196.2	141.3	42.4	67.2	108.4	70.3
**Gender**														
Men	434.3	213.3	46.7	75.5	15.0	26.5^*^	30.5	82.0	183.2	124.2	51.6	73.0^**^	107.3	72.3
Women	426.2	202.9	31.0	65.5	20.7	34.2	19.6	66.6	217.1	163.6	27.6	53.7	110.2	67.4
**Age group, years**														
65–74	423.7	189.2	32.3	51.4	17.9	31.3	33.0	80.4	192.0	133.7	45.8	71.9	102.7	69.8
75–84	438.7	227.6	49.1	87.3	16.5	28.3	19.7	72.1	200.3	148.9	38.9	62.3	114.2	70.7
**Educational attainment**														
≤high school	433.5	204.9	43.3	72.1	15.9	29.4	23.7	80.2	208.1	149.7	33.3	57.3^*^	109.2	74.8
≥university	427.6	216.3	36.7	72.2	19.1	30.4	30.4	70.5	177.5	125.6	56.5	78.4	107.3	63.0
**Marital status**														
single	446.1	202.3	29.1	52.6	16.0	29.2	18.0	70.8	242.9	153.1^*^	33.1	65.2	107.0	65.0
married	428.0	210.8	43.2	75.5	17.4	29.9	28.1	77.7	186.1	136.9	44.4	67.6	108.8	71.6
**BMI, kg/m^2^**														
<25	410.7	201.1^*^	36.6	67.1	14.9	27.5^*^	25.0	73.0	188.1	134.5	44.6	73.5	101.6	64.2^**^
≥25	476.8	220.2	49.8	81.8	22.3	33.9	29.3	84.2	214.2	154.7	37.4	50.3	123.7	80.7
**Chronic conditions**														
0	403.7	207.9	36.1	44.7	16.8	31.5	24.9	66.4	178.6	125.7	42.9	63.2	104.4	65.9
1	430.2	211.1	44.1	75.5	17.7	31.7	31.9	84.1	181.7	122.4	43.5	73.5	111.3	66.6
≥2	450.9	207.7	40.8	83.4	16.9	26.8	22.1	75.9	221.6	163.5	41.0	64.2	108.6	76.9

BMI, body mass index; PC, personal computer; SD, standard deviation; TV, television.

Using multivariate linear regression, the differences in each self-reported domain-specific measures of sedentary behavior between subgroups of each sociodemographic variable was examined with two-sided tests.

^**^P < 0.01, ^*^P < 0.05.

Table 3 shows the findings on the associations of six sedentary domains with objectively-measured prolonged sedentary time (minutes) and number of breaks (per sedentary hour). In the adjusted models with covariates, TV viewing and PC use were significantly associated with longer sedentary time accumulated through the prolonged bouts. Every 10-min increment in watching TV per day was associated with an average 2.9 minutes longer prolonged sedentary time. Also, every 10-min increment in using PC per day was associated with an average 2.2 minutes longer prolonged sedentary time. Longer TV viewing was also significantly associated with less breaks per sedentary hour. Every 10-min increment in watching TV per day was associated with an average 0.06 less breaks per sedentary hours. Longer PC use was associated marginally with less breaks per sedentary hours.

**Table 3.** Relative contributions of sedentary domains to objectively-measured sedentary patterns

	Unadjusted model				Adjusted model^a^			
	
	β^b^	95% CI		*P*	β^b^	95% CI		*P*
**Sedentary time accumulated through prolonged bout, minutes**								
Car	−0.140	−1.936	1.655	0.878	−0.637	−2.262	0.988	0.441
PT	−0.726	−5.125	3.674	0.746	−0.618	−4.636	3.400	0.762
work	0.410	−1.312	2.132	0.640	−0.067	−1.627	1.493	0.933
TV	2.827	1.916	3.737	<0.001	2.928	2.093	3.764	<0.001
PC	3.060	1.123	4.997	0.002	2.231	0.456	4.005	0.014
Other	1.774	−0.071	3.619	0.059	1.614	−0.050	3.278	0.057

**Number of breaks per sedentary hour**								
Car	0.009	−0.036	0.054	0.702	0.027	−0.016	0.070	0.213
PT	0.041	−0.069	0.151	0.464	0.030	−0.077	0.136	0.583
work	−0.017	−0.060	0.026	0.434	−0.011	−0.052	0.030	0.602
TV	−0.060	−0.083	−0.037	<0.001	−0.062	−0.084	−0.039	<0.001
PC	−0.056	−0.104	−0.007	0.024	−0.040	−0.087	0.007	0.096
Other	−0.042	−0.088	0.004	0.075	−0.035	−0.079	0.009	0.117

CI, confidence interval; Other, other leisure; PC, personal computer; PT, public transport; TV, television.

^a^Adjusted for gender, age, body mass index, marital status, educational status, the number of chronic conditions, and accelerometer-wear time (minutes/day).

^b^β: unstandardized coefficients corresponding to 10-minute increment of domain-specific sedentary behavior.

## DISCUSSION

Previous studies on older adults’ sedentary behavior have focused primarily on total time spent in sedentary behavior or a few specific domains of sedentary behavior.^9^^–^^11^^,^^15^^,^^17^ Addressing this gap, the present study examined the patterns and domains of sedentary behavior and the relationships between the pattern and domains among older Japanese adults.

According to the objective measurement, 58% (men: 62%; women: 53%) of daily waking time was spent sedentary in this sample of Japanese older adults. The proportion of time spent sedentary was lower than that found in other accelerometer-based studies: 66% among older women in the United States^11^; 72% among older men in the United Kingdom^10^; 75% among older adults in Iceland^14^; and 72% among older adults living in retirement communities in the United States.^9^ These variations could be partly attributed to different age ranges and compositions of study samples. However, it has been shown that the Active style Pro device used in this study underestimates the amount of sedentary time by 11% in comparison to Actigraph GT3X, which was commonly used in previous studies.^23^ A previous study conducted in Japan using Active style Pro with a sample of a similar age range found that 55% of the wear time was sedentary.^15^ These findings suggest that even considering the measurement properties of the device used, Japanese older adults may be less sedentary in their daily life on average than are those in Western countries.

The patterns of sedentary behavior observed in this study were somewhat similar to those found in previous studies with similar age ranges of participants. Studies on older British men and American women reported 7.2 and 9.0 breaks (7.6 breaks in this study) per sedentary hour, and 5.1 and 3.8 times (4.4 times in this study) of prolonged sedentary bouts, which occupied 43% and 32% of the total time spent in sedentary behavior.^10^^,^^11^ It is possible to argue based on these findings that the way sedentary time is accumulated among older adults may be relatively similar across different countries, whereas the duration of sedentary time along with physical activity breaks may be more sensitive to social, cultural, and environmental differences between countries. There may be between-country differences in daily routines (errands, social activities, recreational activities) for older adults. Since older adults not working tend to spend a longer time at home, differences in housing and local environments may also account for the observed difference in sedentary behavior between countries. It is possible that culture-specific interventions to reduce sedentary behavior may need to be developed and tested.

Men, those of older age, and those who were overweight were found to be more sedentary in terms of total time spent in sedentary behavior and the number/proportion of prolonged sedentary bouts. The findings were generally consistent with those reported in previous studies.^9^^–^^11^^,^^14^ These groups are likely to benefit from interventions aiming to break up prolonged sedentary bouts by taking active breaks. A previous study has shown that older adults tend to have fewer breaks and accumulate a larger proportion of sedentary behavior with prolonged sedentary bouts than do middle-aged adults.^27^ Focusing on breaks may be a promising approach to address sedentary lifestyles among older adults.^28^

The present study examined how time spent in sedentary behavior in older adults was distributed across various domains, including leisure and transport. TV viewing time was found to occupy the largest portion, nearly half, of total time spent in sedentary behavior. This finding was mostly consistent with the previous studies on this topic. A Belgian study examining three sedentary domains (driving car, computer use, TV viewing) found that TV viewing occupied 37% of time spent in self-reported sedentary behavior.^21^ A Japanese study that explored sedentary behavior in five leisure domains found that 52% of time spent in leisure sedentary behavior was for TV viewing.^19^ In addition, the present study found that TV viewing was a major contributor of longer time spent in prolonged sedentary bouts and less frequent breaks during sedentary time, whereas other domains occupied relatively small portion of total sedentary behavior and were not associated with health-risk sedentary patterns in Japanese older adults. Thus, it can be argued that detrimental associations of sedentary behavior while watching TV with various health outcomes^29^ may be due to the continuous nature of TV viewing. Breaking TV time should be a key strategy to reduce the health impact of sedentary behavior among older adults.

Our study found that domain-specific sedentary behavior was associated with different demographic characteristics. For instance, women had longer time spent in sedentary behavior while using public transport. One possible reason would be that older women, who are less likely to have driver’s license than are older men,^30^ may still have to go outside for social activities and errands. Older adults who were single had longer time spent in sedentary behavior while TV viewing. The previous study among Japanese older adults revealed living alone were also significantly associated with prolonged TV viewing time (≥2 hours/day) only in women.^31^ The results from the present and previous studies highlight the need to take sub-groups into account in efforts to reduce domain-specific sedentary behavior.

Some limitations need to be considered in interpreting the present findings. Although a relatively large sample of older adults was initially recruited using random sampling, the final sample size was reduced to less than 300, which may have introduced potential sampling bias. As shown above, the sample retained for analysis was more likely to be married and to be physically active than were those who did not complete the on-site examination, although they did not differ in other demographic characteristics and in the number of chronic conditions. Therefore, the findings might not be applicable to the general older population, in particular to those who were not physically active on a regular basis. Next, self-reported measures were needed to obtain time spent in domain-specific sedentary behaviors, but they could be subject to recall error and social desirability bias. Also, the validity and reliability of this self-reported measure to assess sedentary time in different domains had previously been tested only among middle-aged adults,^26^ which may have led to some inaccuracy of the estimates for the sample in our study. The strength of this study is the use of objective and subjective measures of sedentary behavior, which allowed us to investigate which domains of sedentary behavior contributed more to prolonged sedentary time.

### Conclusions

This sample of Japanese older adults was sedentary for about 60% of their waking hours. Men, those who were older, and those with higher BMI accumulated longer time spent in sedentary behavior through prolonged bouts. TV viewing and PC use were the major contributors of prolonged sitting. For Japanese older adults, initiatives to address sedentary behavior could focus on breaking-up prolonged periods of sedentary behavior by encouraging more frequent breaks, especially during TV viewing.
